# The Vacuum-Assisted Closure Device Increases Value in the Treatment of Gustilo Grade IIIb Open Tibia Fractures in Children

**DOI:** 10.7759/cureus.10194

**Published:** 2020-09-02

**Authors:** Alex Mayers, Mark L Dunleavy, Michael M Chau, William Hennrikus

**Affiliations:** 1 Orthopaedics, Geisinger Medical Center, Danville, USA; 2 Orthopaedics, Penn State Health Milton S. Hershey Medical Center, Hershey, USA; 3 Orthopaedics, University of Minnesota School of Medicine, Minneapolis, USA

**Keywords:** vacuum-assisted closure, value, open, fracture, pediatric, children

## Abstract

Background

Wound management associated with Gustilo grade IIIb open tibia fractures in children often requires muscle flaps, skin grafts, and amputations. The purpose of this study is to report the outcomes and complications of vacuum-assisted closure (VAC) treatment, as well as discuss its role in optimizing value when treating these injuries.

Methods

A retrospective review of medical records and imaging studies was performed from 2008-2015. Six pediatric patients with Gustilo grade IIIb fractures managed with the VAC were identified. The time to treatment, frequency of VAC changes, VAC size, and closure attempts, including muscle flaps and skin grafts, were documented. Fracture fixation methods, the incidence of delayed union or nonunion, as well as the occurrence of deep tissue infection and compartment syndrome were detailed.

Results

Five patients were male and one was female with an average age of 12 years (range 8-15 years). All patients sustained a Gustilo IIIb open tibia fracture and were treated with irrigation, debridement, intravenous (IV) antibiotics, fixation, and a VAC as a wound care adjunct. Three patients required both a muscle flap and a skin graft. One patient required a skin graft. There was one case of deep tissue infection. Three patients were treated successfully with the VAC alone and did not require any flap procedures.

Conclusions

Wound care for Gustilo grade IIIb open tibia fractures in children traditionally involved potentially painful twice-daily dressing changes with solutions such as dilute bleach or iodine. The implementation of VAC markedly reduced the frequency of dressing changes every three days. In the current study, the open wound gradually closed with only a VAC in 50% of Gustilo grade IIIb open pediatric tibia fractures. In summary, the VAC is an adjunct that increases value in the care of pediatric patients with Gustilo grade IIIb open tibia fractures (Value = Outcomes/Cost).

Level of evidence

Therapeutic level IV

## Introduction

Open tibia fractures in children are rare and account for only 2% of all pediatric fractures [1]. Approximately 30% of these are high-energy Gustilo grade III injuries, which can result in infection, compartment syndrome, bone loss, and neurovascular damage [2]. Although the soft tissues in grades I and II fractures can frequently be closed primarily, wound management associated with Gustilo grade IIIb tibia fractures often requires muscle flaps and skin grafts. Delays in coverage of greater than one week have been shown to correlate with worse outcomes [3-4].

Traditionally, twice-daily “wet-to-dry” dressing changes have been employed as a temporary barrier until definitive coverage could be achieved [5-6]. Twice daily dressing changes can cause fear, anxiety, and pain in the pediatric population [7-8]. The vacuum-assisted closure (VAC) device is an alternative and effective method for providing an occlusive dressing, only requiring a change every three days. Prior retrospective data in the pediatric literature supports the use of VAC as a means of reducing infection risk and the need for free flaps following open fractures [9-11]. As a result, VAC treatment may play an important role in maximizing health outcomes while reducing spending, thus improving value in the approach to treating severe pediatric open fractures (Value = Outcomes/Cost) [12]. The purpose of this study is to retrospectively examine the outcomes and complications of VAC treatment in a series of grade IIIb open tibia fractures in children and report its role in optimizing value when treating these injuries.

## Materials and methods

The study was approved by the College of Medicine Institutional Review Board (IRB). A retrospective review of medical records and imaging studies from 2008-2015 was performed. The Gustilo-Anderson classification was used to assess the severity of the open fractures. Type IIIB fractures involve fractures with extensive soft tissue injuries requiring a rotational or free flap for coverage [13-14]. Six pediatric patients with Gustilo grade IIIb fractures managed with a VAC were identified. The time to treatment, frequency of VAC changes, and VAC size, closure attempts, including muscle flaps and skin grafts, were documented. All VAC changes were performed in the OR every three days, which allowed wound inspection and prevented tissue ingrowth into the foam [15]. The VAC suction was set at -125 mmHg for all patients. Fracture fixation methods, the incidence of delayed union or nonunion, as well as the occurrence of deep tissue infection and compartment syndrome, were detailed. Descriptive statistics (frequencies, percentages, averages) were used to qualitatively analyze the data.

## Results

A retrospective review of medical records and imaging studies was performed from 2008-2015 and six adolescent patients with Gustilo IIIb open tibia fractures were identified. Five patients were male and one was female with an average age of 12 years (range 8-15 years). All patients sustained a Gustilo IIIb open tibia fracture and were treated with irrigation, debridement, IV antibiotics, fixation, and VAC as a wound care adjunct. The average time from injury to arrival in the emergency department was 51 minutes (range 26-83 minutes). The average time to the first dose of antibiotic was 77 minutes (range 50 to 110 minutes). All five patients were treated with a combination of cefazolin and gentamicin per hospital protocol, which is consistent with the antibiotic regimen most commonly employed at academic institutions [16]. The average time to surgery was 218 minutes (range 159-333 minutes). The duration of hospital admission averaged 13.7 days (range 5-24 days). The patient who stayed 24 days had additional comorbidities. The average length of follow up was 114.3 weeks (range 41-294 weeks). The average VAC size was 83.9 cm^2^ (range 16 cm^2^ -200 cm^2^). The average number of VAC changes in the hospital was 1.7 (range 1-6). The frequency of VAC changes was every three days under general anesthesia. Three patients required both a muscle flap and a skin graft. One patient required a skin graft. This patient’s injury resulted in one case of deep tissue infection (16.7%). Three patients were treated successfully with the VAC alone and did not require any flap procedure (50%). The wound size for each of these three patients was 16cm^2^, 16cm^2^, and 48cm^2^. Two patients required bone grafts. Four fractures were fixed externally while two were fixed with intramedullary flexible nails. Four patients experienced delayed unions (> 4 months to heal) - all eventually healed. There was no case of malunion, leg length discrepancy >1 cm, angulation >10 degrees, ROM deficit, amputation, or deep pin tract infection.

Table 1 provides the location of soft tissue defects and the number requiring flaps while Table 2 lists descriptive statistics regarding VAC changes.

**Table 1:** Location of soft tissue defects and number requiring flaps

Location	Proximal 3^rd^ Tibia	Middle 3^rd^ Tibia	Distal 3^rd^ Tibia
Number of patients (%)	0	3 (50%)	3 (50%)
Incidence of flaps	-	2	1

**Table 2:** Descriptive statistics regarding VAC changes and resultant length of stay and time to union VAC: vacuum-assisted closure

	Min	Max	Mean
VAC size (cm^2^)	16	200	83.9
#VAC changes in the hospital	1	6	1.7
#VAC changes without anesthesia	0	0	-
Days in hospital	5	24	13.7
Time to union (days)	106	928	271
Duration of follow up (weeks)	41	294	114.3

## Discussion

Gustilo grade IIIb open tibia fractures in children can lead to compartment syndrome, nonunion, and deep infection. The pediatric biology of fracture repair provides advantages over adults, however, including a better periosteal regenerative capacity and soft tissue healing ability [17]. Favorable outcomes can thus be achieved when adhering to evidence-based practices of open fracture management, including immediate IV antibiotics, thorough debridement of contaminated and devitalized tissue, and early fracture stabilization [18]. The management of soft tissue defects is evolving. The current trend is to expedite the timing to definitive closure to reduce infection rates [3-4]. Multiple recent studies have demonstrated the safety of early primary closure in grade I open pediatric both bone forearm fractures [18-19]. In the adult literature, early primary closure in Gustilo grades I, II, and IIIA open fractures has been associated with comparable rates of infection and nonunion as delayed closure [19-20]. For grades IIIB and IIIC injuries, Gopal et al. popularized the “fix and flap” technique in adults, which is a combined ortho-plastic collaborative effort to provide as early internal fixation and flap coverage as possible [21]. Nandra et al. advocated a similar technique in the pediatric population [16]. An effective means of temporary coverage is necessary to prevent infection and maintain the integrity of the soft tissues.

Since the 1990s, VAC has been a valuable adjunct to treating complex wounds as both an occlusive dressing and a wound closure device [22-23]. In a recent retrospective review of pediatric open tibia fractures treated with VAC, Dedmond et al. demonstrated a 50% decrease in the use of free tissue transfers or rotational muscle flaps for coverage [10]. Similarly, Shilt et al. retrospectively reviewed 31 pediatric lawnmower injuries comparing VAC and traditional wound management techniques, finding a 30% reduction in the necessity of flaps [11]. In three of the six cases (50%) in the current study, the open wound gradually closed with VAC, obviating the need for a muscle flap procedure. When examining the distal third tibia shaft fractures specifically, only one of three cases (33%) required a free flap following VAC treatment, which is notable, as this is traditionally an area where flaps are required for coverage.

Multiple studies have shown a decreased bacterial load in wounds treated with VAC as compared to traditional “wet-to-dry” dressings [6,24-26]. Halvorson et al. reported a 5% overall infection rate in open pediatric fractures treated with a VAC, much lower than historical controls [9]. In the current study, only one patient (16.7%) developed a deep infection. This infection was successfully treated with seven days of IV vancomycin and three days of oral trimethoprim-sulfamethoxazole.

According to Porter, value in healthcare is defined as health outcomes per cost. Achieving high value for each patient is a priority of the healthcare system [12]. The use of VAC increases value in the treatment of Gustilo grade IIIb open tibia fractures in children for multiple reasons. The use of VAC reduces the necessity for and/or the size of flaps procedures compared to traditional wound care approaches. The estimated daily cost of commercially available VAC treatment is roughly between $100 and $450 (cost $436 at our institution). This cost can be reduced by using locally developed systems in resource-depleted third world countries [15,27]. In comparison, the cost of rotational and free flaps is estimated to be up to $12,000 and $20,000, respectively [28]. Additionally, VAC treatment has been associated with reduced bacterial loads and lower rates of infection in open fractures. The hospital-related cost of an infected tibia fracture is 6.5 times higher than an uninfected case. Sixty-two percent (62%) of this increased cost is related to longer hospital stays [29]. Thus, the reduction in both the need for free flaps and infection rates can reduce costs and lead to an increase in value.

This study has limitations including the retrospective design and small study population from a single center. Additionally, there was no comparison group to act as a control. Future areas of research should prospectively investigate the utilization of VAC to increase value in the treatment of open pediatric tibia fractures, perhaps by including it as a component of a standardized clinical assessment and management plan (SCAMP) such as the one formulated by Luther et al. to treat pediatric torus fractures [30].

## Conclusions

Gustilo grade IIIb open pediatric tibia fractures are challenging injuries to treat. In our study, VAC increased value by reducing the number and frequency of dressing changes, sedations/anesthetics, flaps, infections, and complications historically seen with high-grade open tibia fractures in children. The utilization of a VAC dressing obviated the need for a flap procedure in 50% of cases. Complications included one infection and four cases of delayed union (> 4 months), although all fractures ultimately healed.
